# Extra-Limites

**Published:** 1842-01-01

**Authors:** 


### EXTRA-LIMITES.

### Abstract of Remarkable Cases, communicated at the last Meeting of the British Association at Plymouth, entitled "Extraordinary Case of Albuminous Ascites with Hydatids; of Hepatic Abscess, with Remarks on the Infrequency of Organic Diseases of the Liver; of Hydatids in that Organ, with extensive Disease under the mask of Rheumatism, and of Phthisis terminating suddenly in Hydrocephalus, also with Co-existent Disease in all the most important Organs of the Body. By Sir *David J. H. Dickson*, M.D. F.R.S. Ed. F.L.S. Inspector of Hospitals, Vice-President of the Medical Section, and of the African Institute of Paris, &c. &c.

First Case of Albuminous Ascites with Hydatids.—*James Prendergast*, pensioner, æt. 46, but in appearance much older, who had been invalided from this hospital, two years previously, and subsequently in Haslar, with ascites, was re-admitted greatly emaciated, and with the abdomen enormously enlarged, on the 11th September last. The dyspnœa and oppression were so great, that, though with little hope of benefit, it was deemed advisable to attempt to relieve him by tapping on the 17th; but about a pint only of a yellow gelatinous looking fluid slowly escaped through the round canula, and he died on the 27th September, 1840.

*Sectio Cadaveris*, thirty-one hours post-mortem.—The abdomen was much distended by a clear semi-concrete matter, nearly resembling half-liquified calves-foot jelly, while a thinner effusion occupied the interstices and viscera, which had been inseparably accreted by pre-existing inflammation of the peritoneal coat; a great quantity of this matter covered the peritoneum, and adhering to its processes, was detached in filmy vesicular masses, and also nearly filled the cavity of the pelvis; other globular bodies were distinctly invested by a fine, pellucid membrane, accurately resembling hydatids, some of them being smaller were appended to larger ones, which were attached by necks to the peritoneal surface of different organs, or in firmer cysts, were embedded in their structure; especially in the spleen. These vesicular cysts were of various sizes from a small grape, or nut, to that of an orange; but they decreased in distinctness of organization as they increased in size. The liver and kidneys were small, rather pale and soft. The stomach, viscera, urinaria, and other organs, appeared to be healthy. The more fluid portion of this jelly-like effusion coagulated by heat, and on being tested by the nitric and acetic acids, tincture of galls, &c. was found to consist principally of albumen with a smaller proportion of gelatin.

The reading of this case, by Sir David, gave rise to some discussion on the nature of false and true hydatids, upon which Professor Williams made some interesting remarks: and another case was subsequently adduced by the writer, where numerous hydatid cysts within each other, were found in an excavated liver, of every size from a pin's head to that of an egg, but the greater number much resembling bunches of the small, white grape, in size and appearance.

Sir David Dickson subsequently presented some cases of hepatic abscess, the contents of two of which made their way though the diaphragm into the bronchi, and were discharged by expectoration; one of them by opening the abscess externally; and another by escaping into the cavity of the abdomen. These cases, and of which the following is a brief account, were accompanied by some remarks on the comparative infrequency of serious organic diseases of the liver, concluding in the following words. "I may add, though in opposition to some high authorities, that occasionally, but not necessarily, or even frequently, have I observed hepatic and splenic disease to be connected, or co-existent. The rather singular coincidence of these cases occurring within a short period, all of which, except the first, occurred in the same quarter, might lead to the inference that such are of frequent occurrence; but my experience, for nearly eighteen years in this hospital, has induced me to form precisely the opposite conclusion. For although so frequently receiving invalids from foreign stations, in the last stage of thoracic and abdominal disease, and although amongst our, consequently, numerous post-mortem investigations, during that period, we have doubtless often met with changes in the size, consistence, appearance, &c. of the liver; and occasionally very interesting instances of various organic diseases; including what has been termed scirrhus, tubera, hydatids, and other morbid growths, yet the aggregate result of our necroscopic researches, abundantly corroborate my previous remarks, in the July number of the Medico-Chirurgical Journal for 1838, and elsewhere, that whatever may be the case with respect to its functional derangements (although even here, I am inclined to believe that it has not seldom, undeservedly borne the blame for contiguous viscera) that organic diseases of the liver are of much less frequent occurrence, than from the writings of authors we should be led to expect."

Case of Hepatic Abscess. *Joseph Gardner*, R.M æt. 28, was admitted on the 12th October, with pain in the right side, cough, nausea, diarrhœa and hectic fever. From the beginning of November, he continued to expectorate large quantities of pus mixed with bloody mucus; but the hæmorrhage though repeatedly very copious and of a fresh colour, was so much restrained by large doses of acetate of lead and opium, that although bringing up such quantities of blood he survived until the 16th December.

*Sectio Cadaveris* 13 *hours post mortem.*—The lungs were healthy and crepitous, except the lower part of the right lobe, which was invaded by hepatic purulent infiltration, and a small portion of it and of the diaphragm which had been attached to it, destroyed by the matter forcing its way upwards into the bronchi. The cavity of the abscess capable of containing at least 24 ounces of fluid, was lined by a flocculent tissue, with but little condensation of the surrounding parts. The gall-bladder, spleen, pancreas, &c. were normal: portions of the mucous membrane of the stomach and ileum appeared congested, the coats of the large intestines seemed thickened throughout, and the mucous surface abraded so deeply in some places, as to lay bare the fibres of the muscular tunic, which could be raised upon the knife.

The writer here noticed another case of abdominal abscess.—*John Alberry*, also admitted in the last stage of debility, with cough, and hectic fever; who after having expectorated enormous quantities of gross pus, which had, likewise, forced a passage through the diaphragm, was discharged quite convalescent. From the report of the case transmitted with him, the abscess, from the vast quantity of matter he had spit up, must have been of great size, and situated behind the peritoneum, below, and between the liver and the right kidney. This man and another previous case, the particulars of which had escaped recollection, ultimately recovered.

*Joseph Blackburn*, S. æt. 30, considered to be labouring under pectoral disease, was admitted on the 22d November 1839, with a swelling in the right hypochondrium, supposed to be caused by protrusion of the liver from the pressure of empyema on that side of the chest. On examination, the liver was found to be large, painful and indurated, with fever, cough, pain of the right shoulder, &c. and the whole abdomen felt tumid and tender. Leeches, purgatives, mercurials, iodine, &c., and afterwards fomentations and cataplasms, when the formation of matter became evident; the tumor advanced rapidly to suppuration and toward the surface, and fluctuation became perceptible. As the patient was sinking under irritative fever, it was opened by an incision on the 5th December, and above a quart of sero-purulent fluid was discharged with great relief. The hectic symptoms rapidly subsided and re-union of the edges of the wound being prevented, a great quantity of sero-purulent fluid of a sickening, offensive odour, but latterly more thick and purulent, was from time to time discharged. This patient gained flesh and improved so rapidly as at one time to give some hopes of his recovery; but the discharge of pus from the external opening continued, profuse, and was generally mixed with much blood; hectic again set in, with great emaciation, and he died on the 5th February, 1840.

*Sectio Cadaveris.* The liver was enormously enlarged, weighing nearly ten pounds and filling both hypochondria; externally it was mottled. The hypertrophied cellular structure was of a pale buff with occasional vascular patches, and interspersed with small abscesses, varying from the size of a pea to that of a shilling. The inferior part of the right lobe which pushed up the diaphragm, was agglutinated laterally and inferiorly to the abdominal wall, omentum, cæcum and kidney, and was excavated by a large abscess, the contents of which had been discharged through the opening made below the ribs on the right side, and were prevented by the adhesions from escaping into the peritoneal cavity. The whole of the organ was softened and infiltrated by small collections of straw-coloured pus, but without cysts, while the large cavity contained a sanious fluid with flakes of lymph, and communicated with the smaller abscesses and gall-ducts, accounting for the bilious-looking matter discharged a short time before death. There was much fatty deposition among the other abdominal viscera, which appeared healthy and strangely contrasted with the external emaciation of the patient.

*James Lee*, S. æt. 35, of a leuco-phlegmatic habit, was admitted 9th October, 1839, with enlargement and tenderness of the hepatic region, pale, sallow skin, white, chalky tongue, and loose, irregular bowels, without fever. The treatment was necessarily much modified by almost constant vomiting and purging; symptoms of dropsy, especially of the limbs, supervened, and he died on the 19th December.

*On Dissection*, the abdomen was found to contain a small quantity of fluid. The liver was large, and the right lobe very globular, with a depression resembling a cicatrix on its convex surface: fluctuation was very perceptible on its posterior part, and on opening it, about three pints of purulent matter, at first brownish, but afterwards thick and primrose-coloured, escaped. The walls of the sac, which were flocculent, and so thin, that rupture would probably have soon taken place, were in contact with the diaphragm posteriorly, and superiorly; and with the right kidney, inferiorly. The pancreas was remarkably hard, but not enlarged; and its duct was pervious. The kidneys were enormously enlarged, and indurated; the right weighing eleven, the left, fourteen and a half ounces, and forming excellent specimens of the degeneration so well described by Dr. Bright. The next hepatic case to be noticed, is that of

*William Jones*, æt. 46, who was admitted on the 13th December, with the abdomen tense, and painful, in the last stage of emaciation, and indeed moribund, and died the following evening.

*On examinatiou*, the intestines were found to be of a florid red colour: the abdominal cavity contained a large quantity of yellow serum, mixed with pus; which was discovered to have escaped from a large abscess in the superior part of the liver; bounded below, by the right kidney, which it had displaced two inches downwards: the interior of the abscess was lined by a cyst of a honey-comb appearance, and towards the abdominal cavity of considerable thickness. Though not altered in shape, the right lobe felt extremely soft, and on section, exhibited numerous other small abscesses with strong cysts, from which straw-coloured pus flowed freely: the gall-bladder was turgid with pale, yellow bile, and another abscess existed between the spleen and left kidney.

The following, which occurred several years ago, are selected from other instances of serious and extensive disease (without being indicated by proportional severity of the symptoms) co-existing in various important organs of the body.

Case of Hydatids.—*Thomas Richards*, S., æt. 25, was admitted on the 28th Feb. 1835, stated to be labouring under acute rheumatism. His chief complaint was of pain in his knees, and limbs generally; but he also evidently laboured under considerable, though ill-defined internal disease, as he had always a quick pulse, anorexia, a foul tongue, and diarrhœa. On one occasion he had been bled with immediate relief, for a pain in the right side, and his sallow countenance indicated hepatic disease; but the great complaint was of muscular pains and a troublesome cough. He died almost instantly, after eating a better breakfast than usual, on the 29th March.

*Necrotomy* presented the ravages of disease to a most extraordinary degree: the lungs on both sides adhered to the costal pleuræ, and contained several abscesses of various sizes, from which matter escaped in the attempt to detach them: the spleen was in a similar state of suppuration; and a communication was discovered to exist between it and the descending colon; but whether pre-existing, or produced by separating them, could not be determined. The left lobe of the liver presented the appearance of a large abscess; the cyst of which adhered by its convex side to the diaphragm; and by the opposite, or concave surface, to the stomach. The sac which was semi-cartilaginous, and contained some matter of a mellicerous appearance, was filled with hydatids of every size, from a pin's head, to that of an egg; but the greater number very much resembled, in size and form, a white grape. Several again contained some small hydatids within each other; and altogether a finer specimen cannot well be imagined. In the right lobe there was a large abscess, containing at least a pound of purulent fluid and communicating with smaller ones by several fistulous openings. The kidneys, as also the stomach, especially towards the cardia, and the enteric mucous membrane in different places exhibited traces of sub-acute inflammation, and the minute vessels injected, resembling fine sea-weed expanded on paper.

CASE OF PHTHISIS SUDDENLY TERMINATING IN HYDROCEPHALUS; AND OF CO-EXISTENT DISEASE TO AN EXTRAORDINARY EXTENT IN MOST OF THE ABDOMINAL VISCERA.—Mr. *George Roberts*, æt 19, was admitted on the 14th September, 1825, with cough, hurried respiration, and symptoms indicating approaching phthisis, with pain occasionally in the lower part of the right side of the abdomen. He became apparently convalescent, and was discharged on the 9th January, in consequence of swelled testicle, to the surgical wards, whence he returned on the 30th, with cough and other pectoral symptoms, considerably aggravated by a fresh cold. In a few days the chest affection diminished in severity, and the head became proportionally affected, with mild delirium, strabismus, &c. and he died with strongly-marked symptoms of cerebral effusion, on the 8th February. This was a very extraordinary instance of metastasis, or transferrence of morbid action from the lungs to the brain, the erethism of the latter organ terminating suddenly in the "waterstroke" of Dr. Golia.

On *dissection*, the brain externally felt pulpy; and on cutting into it, it was found to be very much softened; particularly towards its basis: all the ventricles were completely distended with water; computed to amount to about ℥viij. The foramen monraianum opened so large a communication between the lateral ventricles as to have easily admitted a quill; and the laminæ of the septum lucidum were so much separated, as to afford an excellent view of the fifth ventricle. The lungs adhered everywhere to the costal pleura, and diaphragm; their substance was thickly knotted with unripe tubercles, none being larger than a pea. The pericardium contained ℥iv. of fluid. The abdominal peritoneum adhered to the omentum, and the latter to the intestines; while the mucous membrane was thickly studded with small, firm, granular bodies resembling in size, the tubercles of the lungs. A small sac, containing curdy, scrofulous pus, was embedded in the peritoneum, covering the superior part of the right psoas muscle; and an hydatid the size of a walnut, was found lying loosely in the right side of the abdominal cavity; where he had complained of pain. The intestines themselves were so completely accreted, by pre-existent inflammation of the peritoneal covering, that they could not be separated without rupturing their coats. Strong adhesions existed between the convex surface of the liver and diaphragm, also between it, the stomach, and the arch of the colon, which were connected with equal firmness to the concave surface of the liver. In fine, it was astonishing to witness the extent of peritoneal inflammation and consequent agglutination of the viscera in this case, without its having excited greater uneasiness, or more prominent symptoms.

DAVID J. H. DICKSON, Inspector.

*Naval Hospital, Plymouth*, 5*th Dec.* 1841.

### COPY OF A LETTER

*From Dr.* W. B. Carpenter *of Bristol (England), to Professor* Dunglison *of Philadelphia, in reference to certain Charges made against the former, by Dr.* Martin Paine, *Professor of the Institutes of Medicine in the University of New York, in his "Examination of Reviews, &c."*

*Bristol, Nov.* 16, 1841.

My Dear Sir,—Having just received from Dr. Paine a copy of his "Examination" of the Critique on his Medical and Physiological Commentaries, which appeared in the April Number of the British and Foreign Medical Review, I find, to my great surprise, that Dr. P. has thought himself justified, not only in singling *me* out as the Author of it, and in animadverting upon what he considers to be *its* misrepresentations, as if they were *mine* (thereby attempting to make that a matter of personal discussion between us, for which the Editor of the Review holds himself responsible,)—but also in fixing upon me a charge of literary plagiarism, which is calculated, if I allow it to remain uncontradicted, to do great injury to my personal as well as to my scientific character.

Before going further, I must express my astonishment that any person holding the position which Dr. Paine occupies, should commit himself to so grave a charge against an individual, to whose discredit he *knows* nothing, upon evidence so flimsy as that which he adduces;—especially as he must have been aware that, from the distance of the accused party, his defence could not be laid before the public, until many months should have elapsed since its publication, during which time, an injurious impression would have been formed not easily to be eradicated. And I think that I have further a just right to complain, that Dr. Paine's inculpation of me is not confined to surmise; but that, after he has proved his point to his own satisfaction, he has taken it for granted, and, throughout the latter part of his pamphlet, has continually coupled my name with the accusation of gross plagiary.

The evidence which Dr. P. adduces in support of his charge, is briefly the following. —Having made up his mind, from certain coincidences of opinion and of expression, between the Critique on his Commentaries, and my Principles of Physiology, that I must be the writer of the former, he has searched in previous numbers of the same Review for Articles written, as he imagines, by the same Author. In this search he thinks himself assisted by references occasionally made from one Article to another,—the complete fallacy of which kind of evidence is exposed in Dr. Forbes's letter. Upon the same evidence, I must have been the Reviewer of my own work; and I am not certain whether Dr. P. does not mean to insinuate as much. Any person, however, who carefully reads that review, which I did not see until it was in print, may find abundant evidence of the absurdity of such an idea. With respect to the other chief source of Dr. P.'s evidence,—coincidence in opinion, and in the mode of expressing it, —I will only say that Dr. P. shows great ignorance of the state of physiological science in this country, if he imagines that the opinions expressed in my Principles, on the subjects alluded to, are at all peculiar to myself; and it is very natural that one writer should almost unconsciously adopt the phraseology of another who has recently treated of the same questions, when desiring to express the same ideas.

So much for the evidence on which Dr. P.'s charge is founded. I have thus examined it, merely to show how unjustifiable it was in Dr. P. to charge me with the perpetration of a gross literary theft, upon no better grounds. The charge itself,—that in a review of Hunter on the Blood, in a former volume of the same Journal, I unceremoniously adapted certain passages from Dr. Channing's Essay on Milton, to a very different purpose, is easily disposed of. *I did not write that review.* To those who know me, my simple denial would, I am confident, be amply sufficient; but for the satisfaction of Dr. Paine, who, in his ignorance of my character, may think me as capable of asserting a falsehood, as of stealing a paragraph, I enclose a note from Dr. Forbes confirmatory of my assertion.

Dr. Paine considers that his identification of me with the plagiarist is triumphantly confirmed, by a correspondence which he imagines that he has detected, between certain passages in my Principles of Physiology, and others which he has selected from Dr. Channing's Sermons. I am myself completely at a loss to discover this correspondence; and my friends here find it equally difficult. The falsity of this charge is as easily proved as that of the other; for *I have never* (I speak it almost with shame) *read the Sermons* from which Dr. P. quotes. The ideas which I have expressed, have so long been familiar to my mind, that I cannot imagine that they involve anything peculiarly *Channing-ian.* If any correspondence do exist, it is easily accounted for by the fact, that I received my education from one, who was for many years the respected and attached friend of that illustrious man, and whose mind, cast in the same mould with his, impressed mine with those habits of thought, which have led to whatever similarity may present itself between our published opinions.

In regard to Dr. Paine's criticisms upon the scientific opinions I have expressed in my Principles of Physiology, I shall not now offer any remarks; nor do I intend to take up the gauntlet from an opponent, who has shown himself so destitute of judgment and of good feeling. Of the merits of our respective productions I am quite content to leave the public to judge.

Having few means of placing my statement before the Medical Public of America, save through your mediation, I take the liberty of so far trespassing on your kindness, as to request you to gain insertion for it in such Journals, as may give it a circulation equal to that of Dr. Paine's calumnious charges against me.

Believe me to remain, Dear Sir,

Respectfully and sincerely yours,

WILLIAM B. CARPENTER.

---

*From Dr. Forbes, Editor of the British and Foreign Medical Review, to Dr. W. B. Carpenter.*

DEAR CARPENTER,—As I think it would be a piece of silliness, only second to that of writing and publishing the "Examination," to attempt any detailed or serious reply to Dr. Paine's wordy reclamation, or any justification of the article in the Review to which it refers,—I shall take no notice whatever of his attack, further than relates to the charge of plagiarism. *This is true,* so far as the writer of the review on Hunter is concerned, but *false* as concerns *you,*—since you did not write that review. This I am ready to state to all persons, at all times, as the truth, without any reservation or equivocation. The conduct of the writer of that review, in palming upon the Editor a portion of the writings of another for his own,—if really done intentionally and with a view to deceive (I would fain hope that the fact may admit of some other interpretation), cannot be sufficiently reprobated. Although, as being the first specimen I had had of this person's writing (and, with one trifling exception, the only one I have ever had) I might be forgiven for not suspecting the authenticity of the surreptitious passages, I take shame to myself for being so little acquainted with the eloquent writings of Dr. Channing, as not to detect the theft before the MS. left my hands for the press.

Perhaps when Dr. Paine discovers that he is mistaken in the affiliation of this portion of the Review, he may feel somewhat less confident of the evidence by which he thinks he has traced the authorship of other articles in it to you. I certainly shall not gratify his curiosity on this point, by either affirming or denying the accuracy of his conclusions; and I do not see any reason why you should.

It is singular that Dr. Paine should have been so ignorant of the ordinary mode of conducting a review, as not to know that the reference from one article to another is no proof whatever of the identity of the authorship of the two,—even when this reference is made by the writer of the latter article. But, most commonly, such references are made by the Editor, without any communication with the original writer, in the exercise of the privileges inherent in the office of the great editorial WE.

In looking at the vast accumulation of words in Dr. Paine's pamphlet, I confess that I feel regret that the review of his book (just and accurate as I still hold it to be) was not more favourable; as it is melancholy to think that so much time and pains should have been stolen from tasks of usefulness, and expended in elaborating a work, which, of course, no human being will read, except the author himself, perhaps the writer of the inculpated article, and, alas, the Editor of the Review.

It is lamentable to see how this mortification of Dr. Paine's self-love has clouded his judgment throughout the whole composition of his pamphlet; and this obfuscation is nowhere more conspicuous, than where he attempts to convict you of plagiarizing, in your "Principles of Physiology," from Dr. Channing. The very examples he adduces confute the charge.

Believe me, Dear Carpenter, to be,
Most truly yours,
*Old Burlington Street, Nov.* 15, 1841. JOHN FORBES.

---

### ROYAL COLLEGE OF SURGEONS, IN LONDON.

*December* 16, 1841.

THE President and Council finding it necessary to reprint the List of the Members of the College, early in the ensuing year, request all those members who have not attended to the wishes of the Council, as expressed in the following advertisement of the 8th of April last, to send in the proper statement between the 1st of January and the 1st of February, 1842, on which last day the list will be sent to press.

EDMUND BELFOUR, Secretary.

---

### ROYAL COLLEGE OF SURGEONS IN LONDON.

The Council of the College, desirous of furnishing to the Public a correct List of their Members, request that each Member will be pleased to transmit to the Secretary, between the 1st of June and 1st of July in every year, by letter, a Statement containing his name at full length, address and date of Diploma, in his own handwriting, in order that it may be compared with the Chronological List.

The Council will be further obliged by the Member stating it in a similar manner when he has a Degree in Medicine, or the Licence of the Society of Apothecaries.

The Council will be glad to receive corresponding Statements from the Members of the Edinburgh or Dublin College of Surgeons, practising in England or Wales.

*April*, 8*th*, 1841. EDMUND BELFOUR, Secretary.

---

### ROYAL COLLEGE OF SURGEONS IN LONDON.

All Students of Anatomy and Surgery attending Hospital Practice or Lectures in London, and proposing to be Candidates for the Diploma, are required to Register at the College during the last ten days of January, April and October, the several Tickets for Lectures and Hospital Practice to which they shall have respectively entered:—and no Certificates will be recognised by the Court of Examiners unless they shall correspond with such Registrations.

(By order,) EDMUND BELFOUR, Secretary.

*April* 13, 1840.

---

### ROYAL COLLEGE OF SURGEONS IN LONDON.

*Registration of Members.*

The President and Council in publishing the Corrected List of the Members of the present year, with the date of each diploma, regret that so many Members have omitted to make the return during the months of June and July, according to the form proposed by the Council. They are anxious to explain to the Members that the object of this Annual Registration is to furnish the Judges, Magistrates, Clerks of the Peace, Poor Law Commissioners, Boards of Guardians, and the Public generally, with a correct List of qualified Surgeons, in order to prevent the various impositions which have been practised upon them, by ignorant pretenders and other unqualified persons.

The names of all Members who shall not have registered themselves previously to the months of July 1842 and 1843, will be omitted in the Corrected List of the latter year.

The President and Council particularly wish to intimate to all Public Functionaries, that no Diploma can be genuine, in which there is any erasure, interlineation, or other alteration.

*October* 14, 1841.

N.B.—The Corrected List for 1841, may be purchased at the College for One Shilling.

---

### REGULATIONS OF THE COUNCIL RESPECTING THE PROFESSIONAL EDUCATION OF CANDIDATES FOR THE DIPLOMA. 20th August, 1839.

*Amended, October* 14, 1841.

I. Candidates will be required, in addition to a Certificate of being not less than twenty-one years of age, to bring proof

1. Of having been engaged in the acquirement of professional knowledge for not less than four years; during which period they must have studied Practical Pharmacy for six months, and have attended one year on the Practice of Physic, and three years on the Practice of Surgery, at a recognised Hospital or Hospitals in the United Kingdom*:—three months being allowed for a vacation in each year.

2. Of having studied Anatomy and Physiology, by attendance on Lectures and Demonstrations, and by Dissections, during three Anatomical Seasons or Sessions *extending from October to April inclusive.*

3. Of having attended at least two Courses of Lectures on the Principles and Practice of Surgery, delivered in two distinct Periods or Seasons, each Course comprising not less than 70 Lectures:—And one Course, of not fewer than 70 Lectures, on each of the following subjects, viz. the Practice of Physic—Chemistry—Materia Medica—and Midwifery with Practical Instruction.

II. Members and Licentiates in Surgery of any legally constituted College of

---

* By a Resolution of the Council, on the 7th of November 1839, no Provincial Hospital will in future be recognised by this College which contains fewer than 100 Patients, and no Metropolitan Hospital which contains fewer than 150 Patients.

Surgeons in the United Kingdom, and Graduates in Surgery of any University requiring residence to obtain Degrees, will be admitted for examination on producing their Diploma, Licence, or Degree, together with proofs of being twenty-one years of age, and of having been occupied at least four years in the acquirement of professional knowledge.

III. Graduates in Medicine of any legally constituted College or University requiring residence to obtain Degrees, will be admitted for examination on adducing, together with their Diploma or Degree, proof of having completed the anatomical and surgical Education required by the foregoing Regulations, either at the School of the University, where they shall have graduated, or at a recognized School or Schools in the United Kingdom.

IV. Certificates will not be recognised from any Hospital unless the Surgeons thereto be members of one of the legally constituted Colleges of Surgeons in the United Kingdom; nor from any school of Anatomy, Physiology, or Midwifery, unless the respective teachers be members of some legally constituted College of Physicians or Surgeons in the United Kingdom; nor from any School of Surgery, unless the respective teachers be members of some legally constituted College of Surgeons in the United Kingdom.

V. Certificates will not be received on more than one branch of Science from one and the same Lecturer: but Anatomy and Physiology—Demonstrations and Dissections—will be respectively considered as one branch of Science.

VI. Certificates will not be received from Candidates for the Diploma who have studied in London, unless they shall have registered their Tickets at the College as required, by the regulations, during the last ten days of January, April, and October in each year:—nor from Candidates who have studied elsewhere, unless their names regularly appear in the Registers transmitted from their respective Schools.

N.B.—In the Certificates of attendance on Hospital Practice and on Lectures, it is required that the dates of commencement, and termination, be clearly expressed, and no interlineation, erasure, or alteration will be allowed.

Blank forms of the required Certificates may be obtained on application to the Secretary, to whom they must be delivered properly filled up, ten days before the Candidate can be admitted to examination; and all such Certificates are retained at the College.

---

### MUSEUM.

The MUSEUM is open to the Members of the College, and to the Trustees of the Hunterian Collection, and to Visitors introduced by them personally, or *by written orders stating their names; which orders are not transferable;* on the public days, which are Mondays, Tuesdays, Wednesdays, and Thursdays; from Twelve to Four o'Clock, except during the month of September, when the Museum is closed.

The Museum is open on public days to all Fellows and Licentiates of the Royal College of Physicians in London, to Peers and Members of Parliament, to the Great Officers of State, and of the Royal Household and their immediate Deputies; to all the Dignitaries of the Church and of the Law, to all General and Flag Officers, to the Members of all the Learned and Scientific Bodies in the United Kingdom, to the Members of all the Public Boards, and *to persons*

*introduced personally by them respectively*. And to all respectable Foreigners, and to the Articled Students of the College; on entering their Names and Ranks or Stations in the book provided for that purpose.

Lastly, the Secretary and Conservators will exercise their judgment in giving admission to any respectably dressed Persons, who may apply for it.

The Museum will be open on Fridays to Gentlemen desirous of studying in it, from Twelve to Four in Winter, and from Twelve to Five in Summer, on their making a written application to the President or Museum Committee.

The Senior Conservator, Mr. Clift, will attend every day on the Visitors and Students, and both the Conservators, Messrs. Clift and Owen, on Saturdays from Ten to One, on which day, Visitors and Students desirous of comparing Specimens with those in the Museum, or of having Specimens examined, or of gaining other information, are requested to present themselves.

*N.B.* The Parts of the Catalogue of the Collection, already printed, are to be purchased at the Museum at cost price.

### LIBRARY.

The LIBRARY is open daily, Sundays excepted, to Members and Articled Students of the College, from Ten until Four o'clock, from the 1st of October to the 1st of April; and from the 1st of April to the 1st of September, from Ten until half past Five o'clock.

Members have the privilege of personally introducing a visitor.

Persons, not Members, desirous of admission, must make application, in writing, to the President or Library Committee, specifying their Christian and Surnames, Rank or Profession, and Residence.

Tickets of admission are granted for six months, at the expiration of which time application must be made for their renewal.

Readers, taking extracts from any book, may not lay the paper on which they write on any part of such book; nor may any tracings be taken from any plate without the permission of the Committee.

Books belonging to the College are not to be written upon; and any one observing a defect in a book is requested to report the same to the Librarian.

Readers desirous of consulting works not in the Library are requested to communicate their wishes in writing to the Librarian, in order that the same may be reported to the Committee.

The admission Tickets are not transferable.

Every person upon admission to the Library is required to insert his Name and Address in a book provided for that purpose.

Readers wishing to refer to any book are requested to furnish the Librarian with the Title or Number thereof written on a slip of paper; and to return such Book to the Librarian before quitting the Library.

*N.B.* The Catalogue of the Library is to be purchased at the College at Twelve Shillings the two Parts.

### TRANSACTIONS.

The COUNCIL proposing to publish, in the course of the ensuing year, a Volume, to be entitled

"*Transactions of the Royal College of Surgeons in London,*"

invite from the Members of the College and other scientific Persons, Communications relating to the improvement of Anatomical and Surgical science.

The subjects proposed to be included in this Publication are specified in the following extract from the Ordinances of the College:—

"The Transactions shall consist of

Original Communications on Surgical subjects.

Collegial and Jacksonian Prize Dissertations, deemed of sufficient originality and merit.

Original Memoirs on Human Anatomy.

Original Memoirs on Comparative Anatomy.

Anatomical Monographs of rare Animals, dissected in the Museum of the College.

Explanations of, and Commentaries on, important Preparations in the Museum, with illustrative Plates.

Statistical Reports from Hospitals."

It is requested that Papers intended for publication in this Volume may be transmitted to the President, at the College, on or before the 1st of May, 1842.

---

### STUDENTSHIPS IN ANATOMY.

### *Ordinances.*

1. Three Studentships in Human and Comparative Anatomy shall be instituted; to be held by each Student for the term of Three Years, at a salary of One Hundred Pounds per annum.

2. Candidates shall be Members of the College, under Twenty-six years of age.

3. The Council shall determine annually whether one or more of such Appointments shall take place during the current year; and shall notify its Resolution by Public Advertisement.

4. The Appointment shall be made in the month of June, or as soon after as possible.

5. The Students shall be subject to such Duties and Restrictions as the Council shall from time to time direct: and in case of misconduct shall be liable to dismissal.

### *Regulations.*

1. A Report shall be made to the Council in the month of March of the number of Vacancies, or expected Vacancies, in these Studentships: whereupon the Council shall determine whether any, and what number, of such Vacancies shall be filled up, and shall direct the necessary Advertisements.

2. Candidates shall transmit to the Secretary, on or before the 1st of May, their Applications for the Appointment, together with Certificates of general good character and of fair acquirements in general learning, signed by two qualified Members of the Medical Profession.

3. A Meeting of the Museum Committee shall be held as soon after the 1st May as conveniently as may be, at which the applications of Persons offering themselves shall be examined, and if approved, they shall be admitted as Candidates.

4. The Museum Committee shall determine the mode of ascertaining the Merits of the several Candidates, and shall, after due investigation, report to the Council which of the Candidates in their opinion possesses the highest merit.

5. Students shall attend in the Museum daily (Sundays excepted) from Ten till Four o'Clock, and shall be entirely under the direction of the Conservators, who shall employ them as they shall see fit: and who shall have the power of granting leave of absence when they think proper.

6. In case of misconduct or neglect, the Students shall be liable to be dismissed at any time by the President and Vice-Presidents, who are to report such dismissal, with the grounds thereof, to the next Meeting of the Council.

The President and Council have great pleasure in announcing that, at the instance of the Director-General of the Medical Department of the Army, the Physician General of the Royal Navy, and the Chairman of the Honourable East India Company; the General Commanding the Army in Chief, the Lords Commissioners of the Admiralty and the Court of Directors, have, with the view of promoting the objects of the College, been pleased to place at the disposal of the President and Council an Assistant Surgeoncy in each Service, once in three years, for such of the said Students as may be considered worthy of these honourable distinctions.

---

THE SUBJECT OF THE COLLEGIAL TRIENNIAL PRIZE OF FIFTY GUINEAS,
*Is the Structure and Functions of the Lungs.*

THE SUBJECTS OF THE JACKSONIAN PRIZES OF TWENTY GUINEAS EACH.
For the next Year, 1842, are

*The Comparative Value of the Preparations of Mercury and Iodine in the Treatment of Syphilis,*

AND

*Injuries and Morbid Affections of the Maxillary Bones, including those of the Antrum.*

These prizes to be written for under the following

CONDITIONS.

Candidates to be Members of the College, not of the Council.

The Dissertations to be in English, and to be distinguished by a motto or device, accompanied by a sealed paper containing the name and residence of the Author, and having on the outside a motto or device, corresponding with that on the Dissertation.

Recited Cases to be placed in an Appendix.

Dissertations for the Jacksonian Prizes to be addressed to the Secretary and delivered at the College before Christmas Day, 1842.

Dissertations for the Collegial Anatomical Prize to be also addressed to the Secretary and delivered at the College before Christmas Day, 1842.

The Prize Dissertations, with every accompanying drawing and preparation, will become the property of the College: the other Dissertations and their corresponding sealed papers will be returned upon authenticated application within the period of three years: after which the papers containing the names of the respective Authors will be burned, unopened, and the Manuscripts will become the property of the College.

---

COPY OF A CLAUSE,

In—"*An Act for consolidating and amending the Laws relating to the building, repairing and regulating certain Gaols and Houses of Correction in* ENGLAND *and* WALES."

"4th GEORGE the IVth, cap. 64. [10*th July,* 1823.]"

"XXXIII. And be it further Enacted,—That the Justices in General or Quarter Sessions assembled, shall and they are hereby required from Time to time to appoint a Surgeon, being a Member of one of the Royal College of Surgeons, to each of the Prisons within their Jurisdiction to which this act shall extend; and every such Surgeon shall and is hereby required to visit every Prison to which he shall be so appointed twice at least in every Week, and oftener if necessary, and to see every Prisoner confined therein, whether Criminal or Debtor, and to report to every General or Quarter Sessions the Condition of the Prison, and the state of Health of the Prisoners under his care; and he shall further keep a Journal, in which he shall enter the date of every attendance on the Performance of the Duty, with any observations which may occur to him in the execution thereof, and shall sign the same with his name: and such Journal shall be kept in the Prison, but shall regularly be laid before the Justices for their inspection at every Quarter Sessions, and shall be signed by the Chairman of the Sessions, in proof of the same having been there produced; and shall and may be lawful for the Justices, at every Quarter Sessions after such Appointment, to direct a reasonable Sum to be paid as Salary to such Surgeon, and also such Sums of Money as shall be due for Medicines and other Articles for the Sick."

---

### Extract of a Clause

Of the Act of 6th George the IVth, cap. 50,

*Exempting persons from serving on Juries or Inquests.*

Provided always, and be it further Enacted, That all Surgeons being Members of one of the Royal Colleges of Surgeons in London, Edinburgh, or Dublin, AND ACTUALLY PRACTISING, shall be and are hereby absolutely freed and exempted from serving upon any Juries or Inquests whatsoever, and shall not be inserted in the Lists to be prepared by virtue of this Act as hereinafter mentioned.

---

### ROYAL COLLEGE OF SURGEONS IN LONDON.

Court of Examiners, December 10th, 1841.

Resolved,—That until the 1st of October, 1842, Candidates for the Diploma of this College be admitted to Examination, under the Regulations of the 14th of March, 1835, or of the 25th of June, 1838, or of the 20th of August, 1839, according to the date of the commencement of their Professional Education; and that from and after the said 1st of October, 1842, Candidates will *only* be admitted to Examination under the Regulations of the 20th of August, 1839, as amended on the 14th of October 1841, unless under particular circumstances of apparent hardship, which must be represented by Letter to the Court.

EDMUND BELFOUR, *Secretary.*

---

FINANCES.—*The* RECEIPT *and* EXPENDITURE *of the* COLLEGE *in the Year from Midsummer-day*, 1840, *to Midsummer-day*, 1841, *were as follows, viz :—*

The RECEIPTS amounted to £14158 6*s.* 4*d.* from the following sources of Income, viz.

| | | | |
|---|---|---|---|
| Court of Examiners | £12671 14 0 | | |
| Rent | 37 10 0 | | |
| Fees on Admission to Council and Court of Examiners | 105 0 0 | | |
| Fee on Certificate of Diploma | 5 5 0 | | |
| Incidental; Sale of Lists, Catalogues, etc. | 39 13 0 | | |
| | | £12859 2 0 | |
| Dividends on Investments in Government Securities | | 1299 4 4 | |
| | | | £14158 6 4 |
| Balance in the hands of the Bankers at Midsummer-day, 1840 | | | 727 17 8 |
| | | | 14886 4 0 |

The DISBURSEMENTS amounted to £14503 4*s* 3*d.* divided under the following heads, viz.

| | | | |
|---|---|---|---|
| 1st. COLLEGE DEPARTMENT, including Council, Court of Examiners, Auditors, Diploma Stamps, List of Members, Collegiate Prize, Salaries, Wages, etc. | £6357 12 7 | | |
| 2nd. MUSEUM DEPARTMENT, including Catalogues, Specimens, Spirit, Bottles, Stationary, Salaries, Wages, etc. | 2823 5 11 | | |
| 3rd. LIBRARY DEPARTMENT, including the purchase and binding of Books, Salaries, etc. | 778 0 0 | | |
| 4th. MISCELLANEOUS EXPENSES, including Taxes, Rent, Insurance, Furniture, and Incidental Payments, etc. | 434 6 3 | | |
| 5th. Studentships in Anatomy | 192 7 7 | | |
| 6th. Repairs and Alterations | 238 19 11 | | |
| 7th. EXPENDITURE UNDER DEEDS OF TRUST, including Hunterian Oration, Lectures, and Jacksonian Prize | 99 17 0 | | |
| | | £10924 9 3 | |
| Investment | | 3578 15 0 | |
| | | | £14503 4 3 |
| Balance in the hands of the Bankers at Midsummer-day, 1841 | | | 382 19 9 |
| | | | 14886 4 0 |

SUMMARY.

| | | |
|---|---|---|
| Incidental income | £12859 2 0 | |
| Permanent ditto | 1299 4 4 | |
| | | £14158 6 4 |
| Incidental Expenditure | 11261 14 2 | |
| Permanent ditto | 3241 10 1 | |
| | | £14503 4 3 |

To the Editor of the Medico-Chirurgical Review.

*London, December* 18, 1841.

Sir,—I was much astonished that a London Medical Journal (the Lancet) of which a political Journal, the Times, appears to be the echo, should so impudently and abusively defend the system of "non restraint" in the treatment of all cases of mental alienation, whilst no one has taken upon himself to undeceive the readers of the Lancet, on a system vaunted in opposition to all good sense and truth, by the "Looker on, and Snap, &c." It may truly be said from the extravagance of their language, that one might expect every intelligent and impartial man would do them justice? The public, however, are not always impartial and just, but too often inclined to pronounce in favour of those who trumpet those sonorous words, humanity, philanthropy, &c.

I therefore took it upon me to defend the cause of truth, but I had reckoned without my host. I was ignorant of the principles of my adversaries, I was then unaware that fairness and integrity formed no part in their cause. I sent a reply to "Looker on," which was advertised in the Lancet for insertion the following week, but I suppose in the mean time the "Looker on" had been consulted, and instead of my letter, appeared an announcement diametrically contradicting that of the previous week, by stating that it would not appear in that Journal.

I am informed it is not the first time such means have been employed, and it is unnecessary for me to comment upon them. The public will be their judges, if you will have the goodness to insert in your valuable Journal a copy of the letter which has been refused by the Editor of the Lancet; I would have given it literally, but as I have before said, I was ignorant of the principles of my adversaries, and neglected to preserve a copy, the sense, however, which I give from memory, is unaltered.

*To the Editor of the Lancet.*

"Ne sutor ultra crepidam."

Sir,—In addressing you on a subject which has lately occupied a conspicuous place in the columns of your Journal, permit me to assure you that I am not only perfectly disinterested on the subject I defend, but a stranger in the country which has given birth to opinions so diametrically opposed to each other. I read attentively from one end to the other the lengthy epistle of the "Looker-on," and my first impression was "sunt verba et voces;" on a second reading, I concluded not only that the "Looker-on," whoever he may be, is entirely ignorant of the treatment of mental diseases, but that he has never had any insane persons under his care; and I venture to assert that which I shall prove in proper time and place, that he is acting the part of the cobbler, who thought the leg extremely ill made on being asked his opinion as to the form of the boot. I have so great an antipathy to discussions unfairly conducted—I am so decided an enemy to contests in which one of the adversaries treacherously conceals himself behind a curtain, that on the one hand I am induced to speak to him with severity, but yet with justice, whilst on the other I feel compelled to take up the cause of two experienced and estimable men,* and to become the echo of that one and only opinion which must prevail in the treatment of mental disease. My peculiar position permits me fearlessly to declare my opinions in opposition to those of the "Looker-on" who is unable to draw on me the animadversions of a public always greedy for the "beau ideal," and always the dupe of those who, in their comfortable mansions, seated by a good fire, and after a generous dinner, accompanied by copious libations of the "wine that cheereth," set themselves up as defenders of suffering humanity. Nothing is more easy to a writer unacquainted

* The letter of "Looker-on," was an attack on the Nottingham Report, by Dr. Blake and Mr. Powell.

with his subject than to mislead on words: the "Looker-on" possesses the organ qualifying for this task in an extraordinary degree of development. I am not aware to what degree my own cranium is furnished with such protuberance, but however that may be, I have long since learned to neutralize the influence of this organic tendency, by going straight to facts, and by addressing myself immediately to the men or things I attack, that is to say, I am not possessed of that courage which would lead me to conceal myself by an anonymous name for the purpose of making myself more formidable.

It is a general opinion in my country, and possibly is the same in England, that one secret opponent is more to be feared than ten enemies face to face, or, to make use of an allegory very applicable in this case, the serpent treacherously veiled beneath the leaves is more to be feared than the lion who shews his fine though formidable teeth. In entering on my subject I would first place it in a fair and just position. I shall be concise, for I shall certainly not commence a discussion without proof that my time will not be thrown away. The subject is undoubtedly beyond the reach of ordinary understanding and experience. I may, if hastily judged, be accused of presumption, but it should be understood I will leave the "Looker-on" no door of escape. He must give his name. He must take up the glove I throw down, and come fairly and loyally into the arena. He must bring forward arguments capable of proof, otherwise I should be compromising our noble profession. I should be degrading myself in my own eyes if I could descend to the ground with one who has not the honesty to avow his own name.

One more paragraph, Sir, and I finish a letter which must only be considered as preliminary, and if the "Looker-on" is really a man who has a proper sense of his own dignity, and the importance of the cause he professes to advocate, he will appreciate my motive in endeavouring to lead him to conduct his argument scientifically, and prevent my having the power to apply to him "critiquer est aisé mais juger est difficile." Every man is not an author, and there are many enlightened members of the medical profession, who are unable clearly to define their ideas on paper, and sometimes they express that which is very unlike what they intend to convey, but with me and with every honest man "le sentiment fait tout," and I care little as to the manner in which it is expressed still more in a doubtful case. I would adopt the best sense:—thus whether Dr. Blake and Mr. Powell have given their opinion clearly or not I do not trouble myself to decide, and should never think of amusing your readers with two columns of words on a phrase more or less equivocal.

This unfortunate phrase in the Nottingham Report I read, and I understood or wished to understand that these gentlemen were of opinion *that restraint may be useful and must be necessary in certain cases of insanity, but that the physician ought always to use it with discretion.* This last phrase, Sir, is the burden of the "Looker-on's" letter, while at the same time it represents the actual state of the question relative to the treatment of mental disease.

Moral and physical restraint ought to be in the hands of the enlightened practitioner what poisons and powerful medicaments are with the experienced physician, and what fire and steel are in the hands of the skilful surgeon, "voila ma thèse"—and I do not fear to defend it victoriously against the "Looker-on" upon the above-mentioned conditions. If he has been led to believe that restraint may be abandoned in all cases, he only requires to be undeceived, and in conclusion I repeat I must have a name and not a "Looker-on"—facts and not "verba et voces"—a man of science, and not a "sutor ultra crepidam."

I have the honor to be, Sir,

Your obedient Servant,

CROMMELINCK, M.D.

*One of the Editors of Annales de Medecine, Legale, d'Hygiene Publique et Privée, et de Maladies Mentales.*

